# Supplementary material for: KCND3-Related Neurological Disorders: From Old to Emerging Clinical Phenotypes
Source: Int J Mol Sci. 2020 Aug 13;21(16):5802. doi: 10.3390/ijms21165802 (PMC7461103; doi:10.3390/ijms21165802)
Supplement: Supplementary file 1 [file ijms-21-05802-s001.zip › KCND3-Supplementary Files 12-08/supplementary material kcnd3 per invio RSID 12 agosto.docx]

**Table S1. Main clinical findings in 68 patients with KCND3 variants**

|  | Sex | Age of onset  /Current age | Ataxia | NDev/CI | Tremor | Parkinsonism  Features* | Dystonia | Epilepsy | Myoclonus | Pyramidal signs | Oculomotor signs | PN | Genotype |
| --- | --- | --- | --- | --- | --- | --- | --- | --- | --- | --- | --- | --- | --- |
| Schelhaas Pt1  Pt2 Pt3 Pt4 Pt5 Pt6  Pt7  Pt8  Pt9  Pt10 | F  F  F  M  M  M  M  M  M  M | 30/87  45/83  45/80  45/55  20/48  27/57  31/56  27/51  ND/28  ND/11 | +  +  + f  +  +  +  +  +  +  + | +  ND  +  -  ND  +  ND  +  ND  + f | -  -  -  -  +  -  + f  -  -  + | -  -  -  -  -  -  -  -  -  - | -  -  -  -  -  -  -  -  -  - | -  -  -  -  -  -  -  -  -  - | -  -  +  -  +  -  -  -  -  - | -  -  -  -  -  +  -  +  -  + | + (GEN, Sac Pur)  + (Sac pur)  + (GEN, Sac Pur)  + (GEN, Sac Pur)  + (GEN, Sac Pur)  + (GEN, Sac Pur)  + (GEN, Sac Pur)  + (GEN, Sac Pur)  + (GEN, Sac Pur, Dysm)  + (GEN, Sac Pur) | +  ND  ND  +  ND  ND  ND  ND  ND  ND | c.1054A>C (p.Thr352Pro)  rs397515476 |
| Duarri A Pt 1  Pt2  Pt3  B Pt1 | M  M  F  M | 44/52  55/d80  ND/64  30-35/? | -  + f  + f  + | -  -  -  + | -  -  -  - | -  -  -  - | -  -  -  - | -  -  -  - | -  -  -  - | -  -  -  + | -  -  -  + (Sac Pur, DB Nys) | ND  ND  ND  ND | c.1119G>A (p.Met373Ile)  rs397515477  c.1169G>A (p.Ser390Asn)  rs397515478 |
| Lee A Pt1  Pt2  Pt3  Pt4 Pt5  Pt6  B Pt1  Pt2  Pt3  Pt4  Pt5  Pt6  Pt7 Pt8  C Pt1  Pt2  Pt3  D Pt1  E Pt1  Pt2 Pt3  F Pt1  Pt2  Pt3 | ND  ND  ND  ND ND ND  ND  ND  ND  ND  ND  ND  ND  ND  ND  ND  ND  ND  ND ND ND  ND ND ND | 46/81  35/78  32/57 17/48  20/59  15/32  35/77  50/61  51/61  40/44  30/45  31/32  30/40  24/44  <55/65  <35/39  39/54  35/49  90/d94  <58/63  51/61  40/65  Teens/32  Early teens/26 | + f  + f  + f  + f  + f  + f  + f  + f  + f  + f  + f  +  +  + f  + f  + f  + f  +  + f  + f  + f  + f  + f  + f | -  -  -  -  -  -  ND  -  -  -  -  -  -  -  -  -  -  +sosp  ND  -  ND  -  ND  ND | -  -  -  -  -  -  -  -  -  -  -  +  +  -  -  -  -  -  ND  -  ND  ND  ND ND | -  -  -  -  -  -  +  -  +  -  -  -  -  -  -  -  -  ND  ND  -  ND  ND  ND ND | -  -  -  -  -  -  -  -  -  -  -  -  -  -  -  -  -  ND  ND  -  ND  ND  ND ND | -  -  -  -  -  -  -  -  -  -  -  -  -  -  -  -  -  ND  ND  ND  ND  ND  ND ND | -  -  -  -  -  -  -  -  -  -  -  -  -  -  -  -  -  ND  ND  -  ND  ND  ND ND | -  -  -  -  -  -  -  +  -  -  -  +  +  -  -  ND  +  ND  ND  -  ND  ND  +  ND | + (GEN, Sac Pur)  + (GEN, Sac Pur)  + (GEN, Sac Pur)  + (GEN, Sac Pur)  + (GEN, Sac Pur)  -  + (Nys, Vert Opht)  -  + (Nys, Sac pur)  -  + (Dip, Sac pur)  + (Sac pur)  + f (Int dip, Sac Pur)  + (Nys, Sac pur)  + (Sac Pur)  ND  -  ND  ND  -  ND  ND  + (Sac Pur)  ND | -  -  -  +  -  -  -  ND  +  ND  -  -  -  ND  ND  ND  ND  ND  ND  ND  ND  ND  ND  ND | c.679_681delTTC p.Phe227del rs397515475    c.679_681delTTC p.Phe227del rs397515475  c.1304G>T p.Gly345Val rs797045634  c.1013T>C p. Val338Glu  rs1571939827  c. 1034G>T p. Gly345Val rs797045634  c.1130C>T p. Thr377Met rs1571636501 |
| Duarri A Pt 1 | M | 39/46 | + f | - | - | - | - | - | - | + | ND | ND | c.1348C>T p. Leu450Phe rs150401343 |
| Smets Pt1 | M | 3/10 | + | + f | - | - | - | + | - | - | + (Sac Pur) | ND | c.877_885dupCGCGTCTTC; p.Arg293_Phe295dup No rsID |
| Wang Pt 1 | F | 16/45 | + f | - | - | - | - | - | - | - | + (GEN) | +/- | c.1196G>A p.Ser390Asn rs397515478 |
| Dong Choi Pt1 | F | 17 | + f EA (minute-->hour) | - | - | - | - | - | - | - | + (GEN) |  | c.1291 C > T, p.Arg431Cys rs777183510 |
| Huin A Pt1  Pt2  Pt3  Pt4  Pt5  Pt6  Pt7  Pt8  Pt9  Pt10  Pt11  B Pt1  Pt2  Pt3  Pt4  Pt5 | F  F  M  F  F  F  F  M  M  M  M  M  F  M  M  M | 66/80  15/68  10/60  35/50  16/49  30/39  23/33  3/33  12/30  3/15  3/6  20-30/d88  20/70  10/52  41/46  2/24 | + f  + f  + f  + f  + f  + f  + f  +  +  +  +  + f  + f  + f  + f  + f | +  +  +  +  -  -  +  +  +  +  +  ND  -  +  +  + | -  -  -  -  -  -  -  -  -  -  -  -  -  -  -  - | +  +  -  +  +  -  +  -  +  -  -  ND  +  +  -  - | -  -  -  -  -  -  -  -  -  -  -  -  -  -  -  - | -  -  -  -  -  -  -  + f  + f  + f  + f  -  -  -  -  + | -  -  -  -  -  -  -  -  -  -  -  -  -  -  -  + | +  -  -  +  -  -  -  -  -  -  -  +  -  +  -  - | -  -  + (Nys)  -  -  -  -  -  -  -  + (Nys)  ND  + (Nys)  + (Nys)  -  - | ND  ND  ND  ND  ND  ND  ND  ND  ND  ND  ND  ND  ND  ND  ND  ND | c.679_681delTTC, p.Phe227del rs397515475  c.679_681delTTC, p.Phe227del rs397515475 |
| Kurihara Pt1 | M | <1/30 | + | + f | - | - | + | - | + | + | + (Sac Pur) | ND | c.1150G>A, p.G384S No rsID |
| Paucar Pt1  Pt2  Pt3  Pt4 | M  F  F  M | 18/78  Childhood/65  Childhood/45  18/21 | + f  +  + f  + f | +  +  +  + | -  + f  -  + | +  -  -  - | -  -  -  - | -  -  -  - | -  -  -  - | -  -  -  - | + (Nys, SNP)  + (Nys, Dysm)  + (Nys)  + (Nys, Dysm) | +  -  -  - | c.1130C>T p.Thr377Met rs1571636501 |
| Wang Pt1 | M | 1,6/5 | - | + | - | - | - | + f | - | - | - | - | c.1174G>A, p.Val392Ile rs786205867 |
| Coutelier Pt1 | M | 30 | + f EA | - | - | - | - | - | - | + | + (Sac pur) | - | c.641A>G: p.Lys214Arg rs142744204 |
| Hsiao A Pt1  B Pt1  Pt2 | M  M  F | Infancy/24  10/39  36/d74 | +  +  + f | + f  + f  ND | -  -  - | -  +  - | +  +  - | -  -  - | +  -  - | -  -  - | + (Sac Pur)  + (Slow Sac)  - | -  -  - | c.950G>A p.Cys317Tyr rs1571939905  c.1123C>T p.Pro375Ser rs1571636508 |
| Current case | M | 3/37 | + | + f | + | + | + | + | - | - | + (Sac Pur, Dysm, FIxation instability) | - | c.901T>C p.Ser301Pro  rs79821338 |

d: age of death; EA: Episodic ataxia; NDev: Neurodevelopmental disorders; CI: Cognitive impairment; PN: Peripheral Neuropathy; Nys: Nystagmus; Vert Opht; Vertical Ophtalmolplegia; Sac Pur: Saccadic pursuit; Dip: diplopia; Int Dip: Intermittent diplopia; Dysm: Dysmetria; SNP: Supranuclear palsy

f: first symptom

*Parkinsonism features includes rigidity, bradykynesia and resting tremor
